# Mismatch of Visual-Vestibular Information in Virtual Reality: Is Motion Sickness Part of the Brains Attempt to Reduce the Prediction Error?

**DOI:** 10.3389/fnhum.2021.757735

**Published:** 2021-10-29

**Authors:** Matthias Nürnberger, Carsten Klingner, Otto W. Witte, Stefan Brodoehl

**Affiliations:** ^1^Hans Berger Department of Neurology, Jena University Hospital, Friedrich Schiller University Jena, Jena, Germany; ^2^Biomagnetic Center, Jena University Hospital, Friedrich Schiller University Jena, Jena, Germany

**Keywords:** virtual reality, virtual reality induced motion sickness, VIMS, EEG, effective connectivity, SSQ, transfer entropy, predictive coding

## Abstract

Visually induced motion sickness (VIMS) is a relevant limiting factor in the use of virtual reality (VR) devices. Understanding the origin of this problem might help to develop strategies to circumvent this limitation. Previous studies have attributed VIMS to a mismatch between visual, and vestibular information, causing ambiguity of the position of the body in relation to its surrounding. Studies using EEG have shown a shift of the power spectrum to lower frequencies while VIMS is experienced. However, little is known about the relationship between the intensity of the VIMS and the changes in these power spectra. Moreover, the effect of different varieties of VIMS on the causal relationship between brain areas is largely unknown. Here, we used EEG to study 14 healthy subjects in a VR environment who were exposed to increasing levels of mismatch between vestibular and visual information. The frequency power and the bivariate transfer entropy as a measure for the information transfer were calculated. We found a direct association between increasing mismatch levels and subjective VIMS. With increasing VIMS, the proportion of slow EEG waves (especially 1–10 Hz) increases, especially in temporo-occipital regions. Furthermore, we found a general decrease in the information flow in most brain areas but especially in brain areas involved in the processing of vestibular signals and the detection of self-motion. We hypothesize that the general shift of frequency power and the decrease in information flow while experiencing high intensity VIMS represent a brain state of a reduced ability to receive, transmit and process information. We further hypothesize that the mechanism of reduced information flow is a general reaction of the brain to an unresolvable mismatch of information. This reaction aims on transforming a currently unstable model with a high prediction error into a stable model in an environment of minimal contradictory information.

## Introduction

Virtual reality (VR) is considered one of the most influential uprising technologies in the 21st century. Technology-orientated companies have recognized this trend and have been investing in VR technologies for a decade. As impressive as the technical progress is, one of the most relevant limitations of this technology was and still is motion sickness. For many VR users, the initial euphoria is replaced by severe nausea and discomfort after about 15 min (Nesbitt et al., 2017). Common terms for this VR-induced motion sickness are “cybersickness”, “virtual reality motion sickness” and particularly “visually induced motion sickness” (VIMS).

From a neurophysiological point of view this is quite to be expected. The visually presented illusion of movement of the self (vection) remains without appropriate correspondence in the vestibular and somatosensory systems (sensory conflict theory) (Reason and Brand, 1975). The resulting motion sickness is therefore primarily labeled “visually induced motion sickness” (VIMS) (Hettinger and Riccio, 1992; Howard and Howard, 1994; Stanney et al., 1997). The main symptom of motion sickness is nausea accompanied by vomiting. Usually there are also oculomotor symptoms, signs of fatigue, and disorientation (Golding, 2016). Little known for example is the Sopite syndrome: depression-like symptoms after prolonged episodes of motion sickness (Graybiel and Knepton, 1976). An excellent overview of the mechanisms and reason for motion sickness is provided by Golding (Golding and Gresty, 2005; Golding, 2016). Since we started to use VR environments (VRE) systematically in our lab, we have consistently observed that there is no sudden onset of nausea and discomfort. There appears to be a continuous and gradual onset in which subjects and patients cannot describe exactly what is wrong at the beginning but soon develop a feeling of vertigo and nausea. Previous studies investigating the neurophysiological basis underlying this phenomenon found fluctuations of EEG rhythms especially in lower frequencies while experiencing VIMS (Chelen et al., 1993; Kim et al., 2005; Chen et al., 2010; Arsalan Naqvi et al., 2015; Chuang et al., 2016; Li et al., 2020). Krokos and Varshney (2021) as well as Lim et al. (2021) were recently able to show an increase of Delta (1–3 Hz), Theta (4–7 Hz) and Alpha (8–13 Hz) power in VIMS. However, with increasing exposure time of a person to a constant mismatch but also due to the increase of mismatch caused by contradictory information, the subjective feeling of VIMS intensifies. Little is known about the relationship between the strength of the VIMS and the known changes in the power spectra. Moreover, our understanding of the neurophysiological basis underlying VIMS would greatly benefit from knowledge about how VIMS is associated with changes in the causal relationship between involved brain areas.

Modern neuroimaging methods are in principle well suited to study changes in brain function. However, since VIMS involves body movements in a virtual simulation environment and potential risks include vomiting and drowsiness, EEG studies are preferred for this topic (Kim et al., 2005; Lin et al., 2007, 2013; Li et al., 2020; Lim et al., 2021).

We therefore designed a combined EEG / VR experimental setting that exposes a subject to increasing levels of VR intensity. While monitoring the subjects’ level of motion sickness, we recorded basic differences in the EEG spectrum. Assuming an increasing mismatch in sensory integration in the vestibular, visual, and somatosensory systems, we also studied the information-theoretic measure of transfer entropy (TE) (Schreiber, 2000; Vicente et al., 2011).

## Materials and Methods

### Subjects

In total 14 healthy right-handed subjects (8 male, age 29 ± 3.4 years) without any known neurological or vestibular disorders participated in our study. The sample size of 14 was determined after sample size calculation following our pilot project. All subjects needed to be inexperienced in VR simulation meaning they had not experienced VR technology for more than 60 min. All subjects gave their written consent to participate in our study. Our local Ethics committee approved the study. Table 1 provides the characteristics of the participants.

**TABLE 1:** Participants and individual results of the simulator sickness questionnaire (SSQ).

			**Simulator sickness questionnaire (SSQ) score**			
**Subject**	**Age**	**Sex**	**Baseline**	**First signs**	**Mild**	**Medium/severe**
1	23	f	0	11.2	29.9	56.1
2	26	f	0	15.0	44.9	74.8
3	27	f	0	26.2	37.4	63.6
4	28	m	0	7.5	22.4	52.4
5	28	f	0	18.7	48.6	82.3
6	29	f	0	15.0	49.4	86.0
7	30	m	0	22.4	44.9	78.5
8	30	m	0	7.5	15.0	53.7
9	31	m	0	22.4	48.6	130.9
10	32	m	0	29.9	33.7	52.4
11	32	m	0	22.4	37.4	58.6
12	32	m	0	15.0	33.7	63.6
13	32	f	0	15.0	41.1	93.5
14	37	m	0	22.4	48.6	93.5
mean/std:	28.8 ± 3.4	6f, 8m	**0 ± 0.0**	**17.9 ± 6.8**	**38.26 ± 11.3**	**74.28 ± 24.7**

*Participants’ number, age and gender are displayed in the first three columns. Summarized simulator sickness scores are shown for each subject starting at the baseline. Regarding their general feeling of discomfort, the three categories first signs, mild MS and medium/severe MS were created (please refer to Figure 2 for more details). Bold values display the mean and std in SSQ score in the respective category.*

**FIGURE 1:**
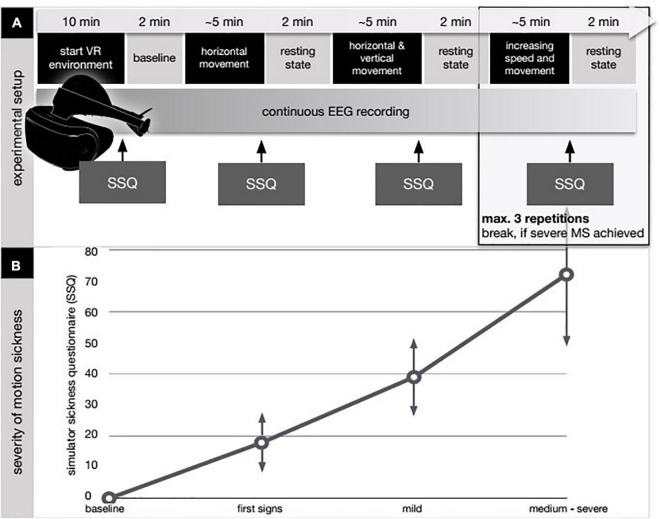
Experimental design and results from the simulator sickness questionnaire (SSQ). **(A)** After applying the EEG cap, the VR device as a head-mounted display was attached and the subjects entered the VR environment (VRE). The first 10 (2 × 5) minutes were spent by getting to know the VRE and the used avatar in the VRE (start VR environment). No EEG was recorded at this point. In the first 5 min the subjects were asked to move their head and their upper body to get used to the environment. In the following 5 min the subjects were not allowed to move while no external movement was applied either thus not to induce motion sickness before the start of the intervention. Next step was to determine a baseline EEG-condition. To achieve this the subjects had to hold still and close their eyes for 2 min (baseline) while EEG was recorded. Afterward, continuous EEG-recording was initiated and the avatar within the VRE was externally moved (movement period). Movement speed and freedom were increased step by step according to a predefined protocol. The subjects had no influence on the movement of their avatar and thus their experienced visual input as they were not allowed to move. After 5 min of movement a resting-state period took place in which the subjects had to hold still and close their eyes for 2 min (resting-state period). After each resting-state period subjects were queried according to the simulator sickness questionnaire (SSQ) for the appearance of sickness-related feelings (Kennedy et al., 1992, 1993; Biernacki et al., 2016; Bimberg et al., 2020). **(B)** Individual results in the SSQ are shown in the bottom diagram. In the baseline condition, the subjects had finished 10 min of habituation in the VR environment. First signs refer to the report of first symptoms after the first movement period. In the mild MS, the subjects reported a beginning discomfort without any signs of dizziness, vomiting, or vegetative agitation (SSQ below 50). Occurrence of those symptoms combined with a SSQ score above 50 was grouped and labeled as medium/severe MS.

**FIGURE 2:**
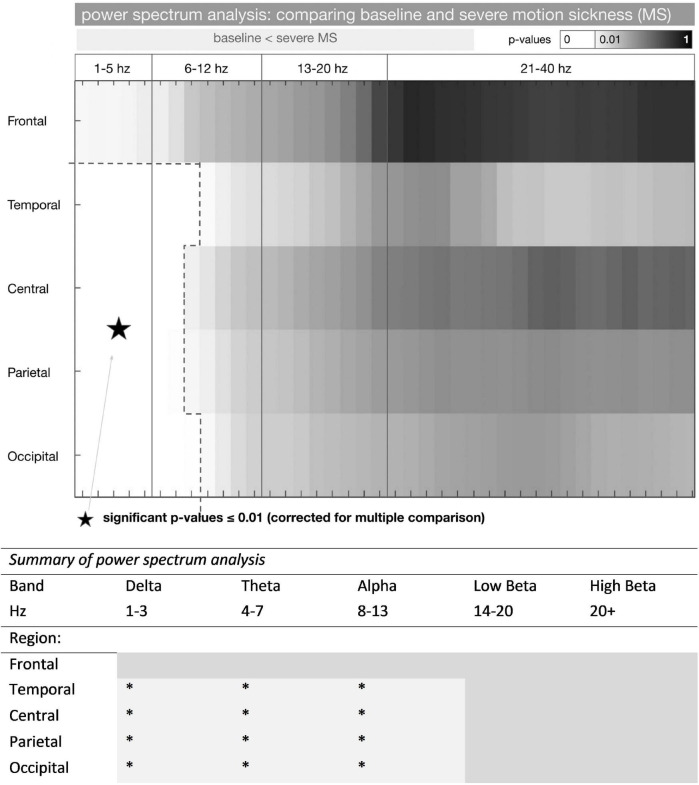
Power spectrum analysis in baseline and severe motion sickness EEG. * represents a significant increase in frequency power (baseline < severe MS) (*p* < 0.01). *P*-values of comparing the standardized mean (mean / standard deviation) of the power spectrum in the baseline and the severe motion sickness EEG are shown (*n* = 14 participants). *P*-values were considered significant at *p* ≤ 0.01 (corrected for multiple comparisons); these frequencies are shown in white. Power spectra for F3/F4, T7/8, P3/P4, and O1/O2 were averaged to frontal, temporal, parietal, and occipital (rows). The frequency separation to 1–5, 6–12, 13–20, and 21–40 Hz derived from a SOM with 2 × 2 groups. A more detailed presentation of these results is shown in Supplementary Figure 1.

### Experimental Design

We applied a multi-stage experiment for this trial (see Figure 1). After applying the EEG cap, the VR device as a head-mounted display was attached and the subjects entered the VR environment (VRE). The first 2 × 5 min were spent by getting to know the VRE and the used avatar in the VRE (start VR environment). No EEG was recorded at this point. In the first 5 min the subjects were asked to move their head and their upper body to get used to the environment. In the following 5 min the subjects were not allowed to move while no external movement was applied either thus not to induce motion sickness before the start of the intervention. Main aspect was to facilitate a first impression of the VRE for the subjects because otherwise a very rapid onset of motion sickness was to be expected. Next step was to determine a baseline EEG-condition. To achieve this the subjects had to hold still and close their eyes for 2 min (baseline) while EEG was recorded. Afterward, continuous EEG-recording was initiated and the avatar within the VRE was externally moved (movement period). Movement speed and freedom were increased step by step according to a predefined protocol. The subjects had no influence on the movement of their avatar and thus their experienced visual input as they were not allowed to move. After 5 min of movement a resting-state period took place in which the subjects had to hold still and close their eyes for 2 min (resting-state period). After each resting-state period subjects were queried according to the simulator sickness questionnaire (SSQ) for the appearance of sickness-related feelings (Kennedy et al., 1992, 1993; Biernacki et al., 2016; Bimberg et al., 2020). This questionnaire derives from the Pensacola Motion Sickness Questionnaire and contains inquiries about 16 sickness related symptoms that must be rated with 0-none, 1-slight, 2-moderate, and 3-severe (Kennedy et al., 1993). Afterward the next movement period was applied. The whole experiment lasted 45 min. First notions of sickness were expected after 15–20 min.

### EEG Data Acquisition

A 18 EEG electrodes with Ag/AgCl electrodes (Fp1, Fp2, F3, F4, F7, F8, Fz, C3, C4, Cz, T3, T4, T7, T8, P3, P4, O1, and O2) were placed according to the 10–20 EEG system. The skin and the reference electrodes were prepared with an isopropyl alcohol swab before calibration. The impedances on all electrodes were calibrated until being less than 10 kΩ. The EEG signals were recorded using a 500 Hz sampling rate. EEG acquisition was accomplished with standard EEG equipment (BrainAmp Standard and Electrode Input Box 64 Channels and ExG Input Box) from Brain Products GmbH (Gilching, Germany). We used the commonly accepted EEG frequency bands Delta (1–3 Hz), Theta (4–7 Hz), Alpha (8–13 Hz), Beta (14+Hz).

### Virtual Reality Environment

For the VR environment, we used a Sony PSVR^®^ head mounted display with a Sony PlayStation 4^®^ system within our EEG lab. Subjects were seated in an armchair and the EEG cap was applied. Afterward the VR device was attached. Subjects entered a tutorial session of a software called *Starblood Arena VR* (v1.0.4^1^). Within this simulator software the subject’s avatar was a small spaceship that could be moved freely in all three dimensions including horizontal and vertical strafing and pitching in the *z*-axis. The environment was experienced in first person perspective from the pilot’s point of view. The subjects were able to move their head to change their view. The movement of the spaceship was controlled by a researcher in a predefined manner in three phases via a PlayStation 4^®^ controller. First phase involved horizontal movement flying in a wide circle through the enclosed area of the above-mentioned tutorial. Second phase included horizontal movement combined with vertical movement flying in a wide circle through the enclosed area while flying up and down on this path. Speed was not changed. Third phase: Horizontal and vertical movement was combined with speed bursts and unexpected directional changes while flying in a wide circle through the enclosed area. Direction of movement was always straight on.

### Data Analysis

All acquired data was analysed on a Windows^®^ 10 (Microsoft, United States) and iMac^®^ i5 (Apple, United States) PC using MATLAB (MathWorks^®^, Natick, MA, United States, Version R2016b) and the Fieldtrip toolbox for EEG/MEG-analysis (Oostenveld et al., 2011). For calculating the transfer entropy, we used the TRENTOOL 3.4.2 toolbox within MATLAB (Lindner et al., 2011).

Preprocessing included manual inspection of the data followed by rejection of artifacts, an ICA for semi-automatized artifact rejection (ECG and EOG artifacts were automatically identified), and down-sampling the data to 300 Hz and bandpass filtering to 0.1–60 Hz. The baseline and resting-state periods were separated into 2 s long parts and a random gap of 0.01–0.05 s. This resulted in 50 to 60 (pseudo-) components for each period called epochs.

Due to bad channel deselection and given that we were mainly interested in interactions of the frontal, central, temporal, parietal, and occipital brain regions, we finally employed the following EEG channels: F3/F4, T7/T8, C3/Cz/C4, P3/P4, and O1/O2.

#### Frequency Analysis

Frequency analysis for the baseline condition and the trials as categorized by the SSQ (the first notion of symptoms, mild motion sickness (MS), medium/severe MS) was performed upon the EEG data. Fast Fourier transformation (FFT) was applied to calculate the absolute power for frequency ranging from 1.0 to 40 Hz. Power spectra of each epoch and condition were averaged.

#### Transfer Entropy

To further evaluate the causal relationship (effective connectivity) of different brain regions we applied the model-free transfer entropy (TE). According to the definition of causality as provided by Wiener (1956) an improvement of the prediction of a time signal X by using the information of the past of a time signal Y can be interpreted as a causal interaction from Y to X. These causal interactions are often referred to as effective connectivity (Friston, 2011). Causal interactions might help to interpret the information flow between different neuronal structures whereas measures of functional connectivity (such as coherence) reflect inherent statistical covariate relations between time-series signals.

By its nature, TE incorporates dynamical asymmetric information transfer and is based on the transition of probability. Moreover, TE can be reformulated as conditional mutual information (Schreiber, 2000). TE is especially well suited for EEG and MEG analysis on the sensor level to reduce signal-cross-talk and identify non-linear interactions (Schreiber, 2000).

We applied the MATLAB toolbox TRENTOOL 3.4.2 for TE calculation of the EEG data (Lindner et al., 2011). Initially, we used a prediction time u of 1–50 ms. Retrospectively the optimal interaction delay was found between 4–12 ms. The TE was calculated pairwise for fixed channel combinations.

#### Group Analysis

For group analysis of frequency power, connectivity, and cross-frequency interactions we used a non-parametric permutation test (number of permutations 5,000, *p* ≤ 0.01, corrected for multiple comparisons by using Bonferroni methods).

To cluster results from the frequency power, we performed a self-organizing-map (SOM) using the Neuronal Network Toolbox^®^ within MATLAB upon the frequencies.

## Results

### Simulator Sickness Questionnaire

At baseline none of the subjects experienced any symptoms of motion sickness. First signs of motion sickness mainly consisting of a feeling of slight general discomfort were reported by all subjects after the first period of virtual movement within the VRE (first 5-min movement period).

A score greater than 0 on the SSQ after a VR movement period thus was labeled as first signs. Here, all subjects scored between 1 and 30 points (mean 17.9 ± 6.8). The subsequent run with an increase in the SSQ score was labeled as mild motion sickness (SSQ ≤ 50, mean 38.26 ± 11.3). In the further investigation, the individual scores diverged as expected. According to the test protocol, the examination ended when motion sickness was subjectively rated as severe and unpleasant or a total of 6 passes in VRE had been completed. Of the 14 subjects, 11 described severe motion sickness symptoms in the final pass, whereas three reported only moderate symptoms in the final pass. In the following analysis, the last phase was therefore referred to as moderate/severe motion sickness (SSQ 50+).

Please refer to Table 1 for individual SSQ and Figure 1 for a summary of our results.

### Frequency Power Analysis

#### Comparing Baseline and Severe Motion Sickness

For the group difference, frequency powers of EEG channels were averaged according to the following scheme: F(frontal) = mean(F3, F4), T(emporal) = mean(T7, T8), C(entral) = mean(C3, Cz, C4), P(arietal) = mean(P3, P4), O(ccipital) = mean(O1, O2).

To cluster the results in the frequency dimension, we initially performed a self-organizing map (SOM) with 2 × 2 nodes upon the frequency power results within 1 to 40 Hz throughout all channels (using the MATLAB Neural Network Toolbox^®^ with standard settings). Thereby the frequencies results were divided according to their expression pattern into four groups: 1–5, 6–12, 13–20, and 21–40 Hz. Given that the same grouping was found in the subsequently performed coherence analysis (results not shown), we did use this clustering throughout the whole data analysis.

The total group difference between the baseline and the severe MS condition is shown in Figure 2. The standardized mean difference was tested for significance with non-parametric t-statistics (5,000 permutations). The group difference was regarded as significant at *p* ≤ 0.01 (*p*-values were corrected for multiple comparisons by dividing them through the number of frequencies (40)).

In summary, we found a pronounced increase of delta and theta activity as well as alpha activity in all EEG channels, except for the frontal electrodes. The increase was most pronounced in the temporal (T7/8) and occipital (O1/O2). For frequencies above 11 Hz, there was no significant difference found.

Correlating the intra-subject difference between the [baseline – severe MS condition] and the SSQ, the difference increased significantly with a larger score in the SSQ (shown by the negative Pearson correlation coefficient in Supplementary Figure 1). Here the strongest correlation was found with the frontal electrodes.

#### Motion Sickness Dependent Changes of the Frequency Power

By calculating a mean power according to the four groups of frequencies, we could determine the average effect of the motion sickness condition. Results are shown in Supplementary Figure 2.

Within theta and delta rhythm, there is a linear increase of the mean power with the severity of MS. For 6–12 Hz, the largest power increase appears with severe MS. For 13–40 Hz, there is no significant difference in the power between the baseline and the medium/severe MS condition. There is, however, a pronounced decline in power at the mild MS condition that is significant for the frontal (F3/F4) and temporal (T7/T8) locations.

### Analysis of Information Transfer

**FIGURE 3:**
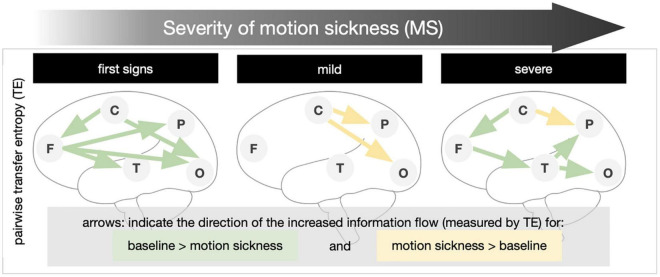
Transfer entropy in levels of increasing motion sickness (MS). Pairwise transfer entropy (bivariate TE) was calculated for the difference of the baseline to (1) first signs of MS, (2) mild MS, and (3) medium/severe MS. The direction of the increased information transfer in comparison to the baseline condition is shown by arrows (results significant at *p* ≤ 0.05 in non-parametric permutation t-statistics).

Transfer entropy (TE) is a method to identify the amount of causal influence a time-series signal exerts on a second signal. We compared the change of the information flow of the conditions with varying levels of MS from the baseline. Pairwise TE calculations are shown in Figure 3. Results of the pairwise TE calculation are shown in Figure 3. With the first notion of MS the information flow from frontal to temporal, parietal, and occipital regions is decreased (green arrows). There is also a decreased influence of the central regions upon the frontal and occipital areas. With mild MS the central regions exert more influence upon parietal and occipital regions (yellow arrows). The transition to medium/severe MS is characterized by a decreased influence of frontal upon temporal, temporal upon parietal, and occipital and central upon frontal regions; the information flow from the central to the parietal regions remains increased.

## Discussion

In our study, we investigated the influence of increasing levels of motion sickness (measured by SSQ) on the frequency power and the relationship between EEG signals. The experimental settings in a virtual reality environment (VRE) were designed to induce motion sickness (MS) by delivering a mismatch between visual and vestibular information.

First, we want to highlight our main findings.

– All participants experienced typical symptoms of MS with increasing intensity of movement in the VR environment (i.e., vection).
– Relative to a baseline EEG (in VR) the power spectrum for frequencies below 10 Hz is increased in all brain regions. The increase in frequency power was correlated positively to the level of motion sickness. Subjects with the highest SSQ had the highest power gain in the theta, delta, and alpha frequencies.
– The flow of information emanating from frontal and central networks is reduced during the *first signs of MS*. In *mild MS*, the modulatory influence of central areas on the parietal and occipital increases. Finally, in *severe MS*, the influences of central upon frontal, frontal upon temporal, and temporal upon parietal and occipital are reduced, while the modulation of parietal areas by central increases (Figure 3, bottom).

### Motion Sickness in Virtual Reality Environment

Symptoms of motion sickness can be classified along the dimensions of nausea, general discomfort/disorientation, and oculomotor dysfunction (Kennedy et al., 1992). A percentage breakdown of all individual outcomes resulted in a distribution of 40:40:20 (nausea:disorientation:oculomotor dysfunction) in our data. Kennedy and colleagues described (Kennedy et al., 2010) that different causes of VIMS, such as seasickness and simulator sickness, have different distributions of these three categories. Thus, the clear emphasis on nausea and disorientation in our results is similar to the profile of space sickness (Kennedy et al., 1994). A significantly higher proportion of oculomotor symptoms can be found, for example, in simulator sickness (e.g., helicopter simulator) with a moving base and many monitors (Stanney et al., 1997; Drexler, 2006). Similar to our findings, Stanney et al. (2003) found a linear trend of increasing SSQ with exposure time.

Vection, the perception of self-motion by stimulation in the visual system (Dichgans and Brandt, 1978; Riecke and Schulte-Pelkum, 2013), is considered the main cause of VR environment sickness (Kennedy et al., 2010). However, vection is also responsible for a relevant proportion of the feeling of presence and immersion in a VR environment (Keshavarz et al., 2015). Interestingly, although humans with bilateral vestibulopathy can perceive vection, they are immune to nausea evoked by vection (Johnson et al., 1999). Even age and sex must be considered regarding the susceptibility to motion sickness (Stanney et al., 2003). For example, children under 2 years of age seem not to experience motion sickness at all. Susceptibility to motion sickness starts at 6 years of age and reaches a peak at 9 years (Reason and Brand, 1975; Turner and Griffin, 1999).

### EEG in Virtual Reality Induced Motion Sickness

Virtual reality motion sickness induced changes upon the frequency powers in EEG are commonly reported (Min et al., 2004; Kim et al., 2005; Jensen and Colgin, 2007; Lin et al., 2007; Park et al., 2008; Chen et al., 2010; Chuang et al., 2016; Li et al., 2020; Krokos and Varshney, 2021). Conversely, the exact patterns of these changes are inconsistent and open to debate. Some authors describe more generalized changes with increased power in the lower frequencies (delta, theta, and alpha) (Min et al., 2004; Jensen and Colgin, 2007; Li et al., 2020; Krokos and Varshney, 2021), whereas other studies revealed more distinctive patterns mainly in temporo-frontal brain regions (Chelen et al., 1993; Park et al., 2008; Chen et al., 2010). In our study, we did observe a significant increase in the frequency power including the theta, delta, and lower alpha spectrum. Background for the differing findings is certainly on the one hand the different experimental settings. Motion sickness was evoked by either visual, vestibular or simultaneous stimulation (Chelen et al., 1993; Wood et al., 1994; Jensen and Colgin, 2007; Park et al., 2008; Chen et al., 2010). Besides, there were passive and very active test conditions in the VR environment (Min et al., 2004; Kim et al., 2005). For example, Chen et al. (2010) used a moving VR simulator platform. In our study, we used passive stimulation, where subjects had to sit still, and their VR avatar was moved remotely. However, in line with the available literature our study demonstrated the general trend of increased frequency power due to increasing levels of motion sickness in lower frequency bands. The fact that alpha and theta in the frequency range of 6–12 Hz have been combined by using a self-organizing map analysis suggest a very similar behavior with increasing synchronization at increasing levels of motion sickness.

### Effects of Dizziness and Drowsiness

An increase in alpha power is found in certain conditions such as fatigue and dizziness; in particular, a recent review summarizes power spectral density changes especially in the alpha spectrum in different vigilance and general conditions (Ismail and Karwowski, 2020). Therefore, it must be argued that changes in frequency power are due to reduced vigilance because of exertion in the VR environment. Changes in the alpha spectrum and below can also be observed with an increase in phasic alertness and attentional demands (Klimesch et al., 1998). Also, changes in central and occipital regions are particularly noticeable during stress (Ray and Cole, 1985; Chen et al., 1989).

### Sensory Processing and Multi-Sensory Integration

Oscillations and synchronizations in the alpha spectrum and below are increasingly understood as an integral part of neuronal communication and integration. Theta oscillations serve to coordinate different brain functions (e.g., an update of motor planning after somatosensory input – sensorimotor integration hypothesis) (Bland and Oddie, 2001). Jensen and colleagues (Jensen and Colgin, 2007) describe that theta oscillations can serve as a kind of carrier wave for information transfer between regions. Results of a further study that performed a wayfinding task in an VR environment suggested that the mechanism of sensorimotor integration is guided by theta oscillations (Caplan et al., 2003).

Also, the alpha rhythm is sensitive to sensory stimulation or the lack of it. In this context, the initially contradictory observation that alpha synchronization is accompanied by inhibition of itself provides a crucial component as the temporal and spatial encoding of alpha activity can effectively integrate neural information in a specific pattern (Klimesch et al., 2007). Accordingly, the observation of increased alpha and theta activity in VR environments is often understood as a multi-sensory integration process (Kim et al., 2005; Barry et al., 2007; Chen et al., 2010; Chuang et al., 2016; Li et al., 2020). In particular, when multi-sensory and distributed networks communicate, this mechanism serves as a top-down controller to coordinate processing demands (Benedek et al., 2011). In short, modulation of the alpha rhythm reflects a top-down modulation that initiates inhibition. This explanation is also well grounded in the theory of predictive coding. The principle of predictive coding suggest that the human brain provides internal models representing different environments. Actions of the body create a prediction of the sensory outcome. Only sensory information that contradict these predictions are further processed and incorporated into the internal model. It is well accepted that cortical oscillations especially at low frequencies play a crucial role in predictive coding (Arnal and Giraud, 2012; Bastos et al., 2012; Sauseng et al., 2015; Alamia and VanRullen, 2019). Accordingly, Sauseng and colleagues (Sauseng et al., 2015) were able to describe slow oscillations in the EEG as an expression of predictive coding in the visual system. Particularly slow delta-theta oscillations are involved in the temporal component of sensory integration (Cravo et al., 2011). Interpreting our findings in this framework suggest that an increase in slow frequencies with sustained exposure to the VR environment reflect an attempt to update the internal model. However, due to the nature of the mismatch information in VR, any attempt of the brain to adapt the internal model must fail and cannot reduce the prediction error.

Following this argumentation, we therefore suggest that our finding of a general shift of frequency power and the decrease in information flow in high levels of VIMS indicate a brain state of reduced ability to receive, transmit and process information. This mechanism was previously suggested as important to establish highly selective activation patterns (Klimesch et al., 2007). We speculate that the mechanism of reduced information flow is a general reaction of the brain to an unresolvable mismatch of information to transport a currently unstable model with a high prediction error into a stable model in an environment of minimal (contradictory) information.

### Prediction Error May Not Be Resolved in the Case of Virtual Reality-Induced Motion Sickness

A fundamental idea in the model of predictive coding is that a stimulus will generate a specific response in the cortex. However, it is not the stimulus itself that is dealt with, but the deviation from the internal model – the prediction error. For further processing and communication with higher levels of the hierarchy only the prediction error is needed (Friston, 2005; Klingner et al., 2016).

In the analysis of the time series of the resting-state EEG, we used the information-theoretical measure of transfer entropy. Bivariate transfer entropy is known to be an effective measure of effective brain connectivity, overcoming the shortcomings of classical analysis methods of functional connectivity (Friston, 2011; Brodski-Guerniero et al., 2017).

Combining the concept of predictive coding with the idea of information flow within systems, it can be inferred that the predictive model must be stored as information in the overall system (Brodski-Guerniero et al., 2017). To derive a prediction from sensory inputs, this knowledge must be contained a-priori within neural signal patterns. Therefore, it should be quantifiable by TE.

**FIGURE 4:**
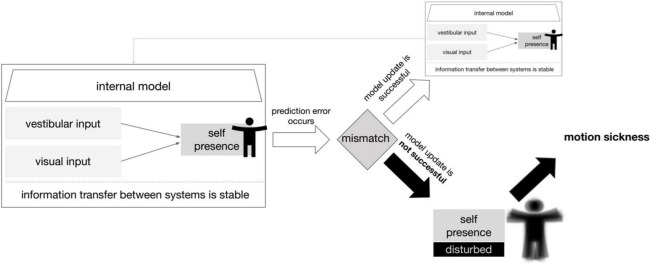
Model of prediction error leading to motion sickness. A simplified scheme in which an update of the internal model is not possible due to a prediction error caused by the vestibular and visual inputs. The consequence for the organism is motion sickness. The term self-presence here describes the ability to perceive the self in physical space including motion in reference to Baumgartner et al. (2008).

We, therefore, formulated the hypothesis that sensory conflict during the VR experience produces a prediction error in the representation of self-motion. Instead of a successful update of the model, there occurs an increasing decompensation of the overall system. Subsequently, this results in a reduction of the information flow within the system accompanied by an increasing discomfort and motion sickness. The overall idea of this hypothetical model is shown in Figure 4.

As expected, evaluation of the TE showed a decrease in overall information flow in both beginning and severe MS. Surprisingly, however, in between (in mild MS) there was a re-establishment of the information flow and even an increase (compared to the rest condition) in centro-temporal and centro-occipital communication (Figure 3). We think that this re-increase is a sign of adaptation processes and an attempt to update the prediction model (Brodski-Guerniero et al., 2017). Ultimately, however, this mechanism fails, and the system collapses at least partially. In comparison with the initial condition and the final stage (severe MS), frontal and occipital regions show a relatively stable information flow. Especially temporal and parietal communication influences are reduced. However, concerning the clinical manifestation of motion sickness, it can only be speculated to what extent the observed changes in brain activity and connectivity are cause or consequence.

### Why Does Visually Induced Motion Sickness Occur in the First Place?

Motion sickness is unpleasant. However, under certain circumstances it can also have devastating effects on the performance and even the survival of an organism. Typically, complex tasks and tasks with sustained attention are particularly affected (Hettinger and Riccio, 1992). Simple tasks are less affected, whereas newly learned skills with spatial orientation involvement are particularly compromised (Golding and Gresty, 2005).

A frequently and controversially discussed point is why motion sickness exists at all. The vestibular system serves facilitates orientation and balance as well as stabilizing the gaze. The fact that other mammals such as rats and also fish can develop forms of motion sickness suggests that this (dys-)function is deeply entangled in our phylogenetic roots (Reason and Brand, 1975; Oman, 1990). Golding provides an excellent overview of current hypotheses (Golding, 2016). For example, an intriguingly simple explanation is offered by the toxin detector hypothesis (Treisman, 1977; Golding, 2016). According to this theory motion sickness might simply be an ancient protective mechanism to detect neurotoxic effects on vestibular, visual, and kinesthetic perception. An even more simplistic approach is offered by Oman (2012). In the evolutionary maladaptation hypothesis, motion sickness is only an unfortunate consequence of the close anatomical entanglements of the motion detection system (i.e., the vestibular system) and the vomiting system in the brainstem (Oman, 2012).

## Conclusion

We have demonstrated that VR-induced motion sickness is associated with distinct changes in brain function and connectivity. Here, we proposed the mismatch of visual information in the absence of adequate vestibular stimulus as a major cause according to the model of predictive coding. It remains to be speculated that the resulting motion sickness is more likely to be an immediate by-product, accessing the phylogenetically ancient vomiting system in the brainstem. Differentiation which changes in brain activity is due to the sensory conflict or caused by motion sickness should be investigated in further studies. Given the increasing importance of VR, a profound understanding of the constraints imposed by VIMS will be increasingly important. Measures to counteract the occurrence of MS or assist in detecting it at an early stage will undoubtedly improve the progress with this promising technology.

## Data Availability Statement

The raw data supporting the conclusions of this article will be made available by the authors upon request, without undue reservation.

## Ethics Statement

The studies involving human participants were reviewed and approved by the Ethics Committee Friedrich Schiller University Jena. The participants provided their written informed consent to participate in this study.

## Author Contributions

MN and SB performed the measurement. MN, SB, and CK performed the analysis. MN, SB, CK, and OW wrote the manuscript. All authors contributed to the article and approved the submitted version.

## Conflict of Interest

The authors declare that the research was conducted in the absence of any commercial or financial relationships that could be construed as a potential conflict of interest.

## Publisher’s Note

All claims expressed in this article are solely those of the authors and do not necessarily represent those of their affiliated organizations, or those of the publisher, the editors and the reviewers. Any product that may be evaluated in this article, or claim that may be made by its manufacturer, is not guaranteed or endorsed by the publisher.

## Footnotes

1
https://whitemoondreams.com/games
