# Supplementary material for: Mismatch of Visual-Vestibular Information in Virtual Reality: Is Motion Sickness Part of the Brains Attempt to Reduce the Prediction Error?
Source: Front Hum Neurosci. 2021 Oct 29;15:757735. doi: 10.3389/fnhum.2021.757735 (PMC8586552; doi:10.3389/fnhum.2021.757735)
Supplement: Supplementary file 1 [file Data_Sheet_1.docx]

# Supplementary Information

## Figure 1

*Frequency power comparing baseline and severe MS*

**
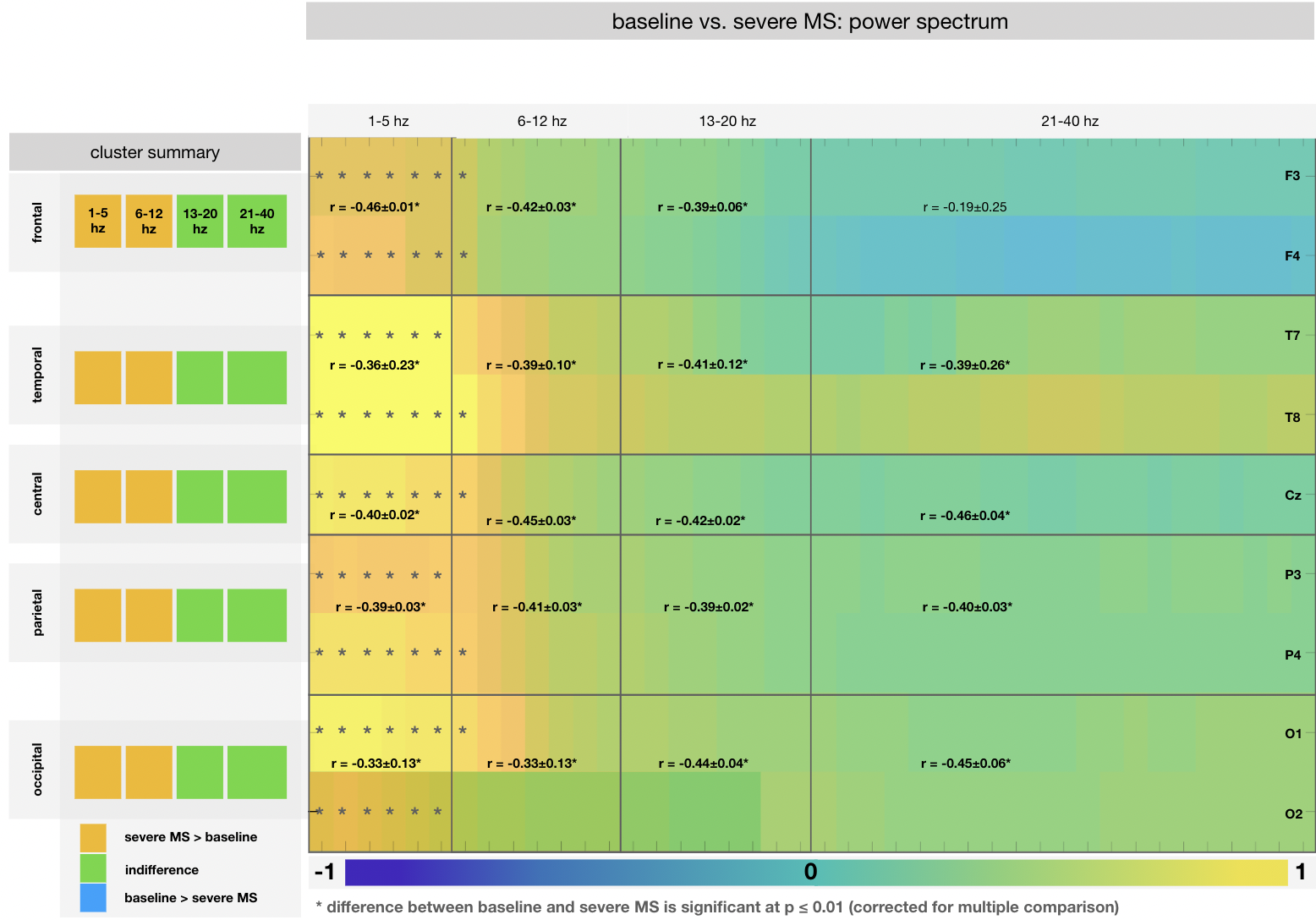
**

Standardized mean (mean/standard deviation) of the power spectrum comparing the baseline and severe MS of all participants (n = 14). The results are grouped for EEG channels (rows) and frequencies (columns). The grouping of the frequencies results from a SOM with 2 x 2 groups. On the left side, a summary of each block (channel and frequency group) is shown, where yellow indicates an increased power in the severe MS and blue indicates increased power in the baseline condition. Star symbols (*) indicate significance in non-parametric paired t-statistics at p ≤ 0.01. For each block (channel and frequency group) the correlation of the difference ([baseline – severe MS]) and the individual simulator sickness score (SSQ) is given (r - Pearson correlation).

## Figure 2

*Frequency power in increasing levels of motion sickness*


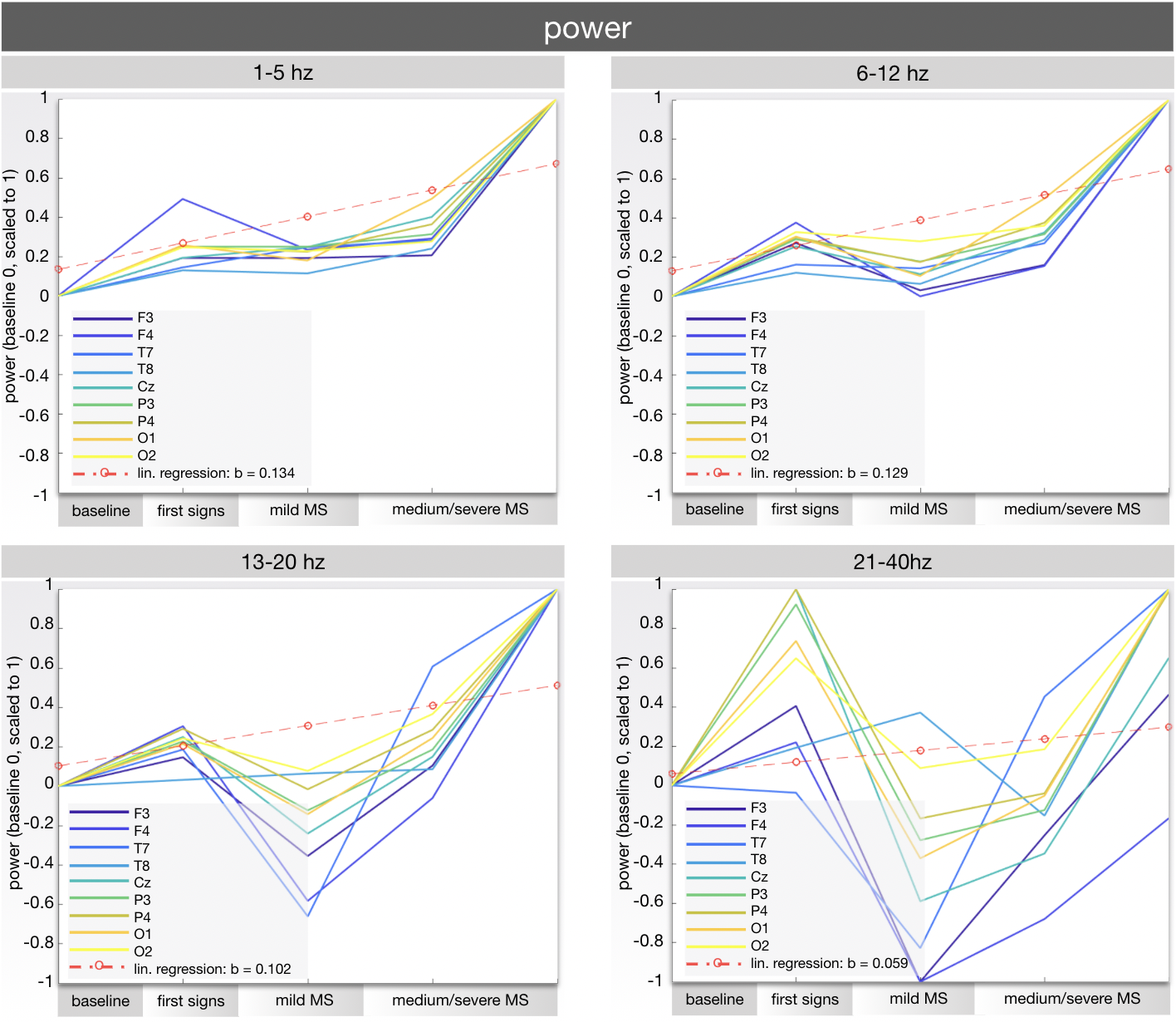


The figure of the frequency power at increasing levels of motion sickness. The 4 frequency ranges from 1 – 5, 6 – 12, 13 – 20, and 21 – 40 Hz are shown; the grouping of the frequencies was taken from the SOM clustering (cf. Figure 2). The values per group are scaled to the interval [-1 1] for better visualization. The slope b of linear regression is plotted by the red line.
